# Adherence and barriers to Mediterranean diet in Tunisian patients with nonalcoholic fatty liver disease

**DOI:** 10.2144/fsoa-2023-0140

**Published:** 2024-05-20

**Authors:** Ayadi Shema, Trad Nouha, Krifa Nesrine, Mensi Asma, Belhadj Mabrouk Emna, Mouelhi Leila, Dabbeche Radhouene

**Affiliations:** 1 Gastroenterology Department, Charles Nicolle Hospital, Faculty of Medicine of Tunis, University Tunis El Manar Tunis, Tunisia

**Keywords:** barriers, mediterranean diet, nonalcoholic fatty liver disease, nutrition, patient compliance

## Abstract

**Aim:** The burden of non alcoolic fatty liver disease (NAFLD) is globally increasing. While crucial for management, NAFLD patient adherence to the Mediterranean diet (MD) is underexplored, especially in Mediterranean countries such as Tunisia. **Materials & methods:** A prospective study (Nov 2022–Feb 2023) supervised MD introduction by a nutritionist, evaluated adherence with MEDAS scores (≥10 for good compliance), and explored barriers to good adherence. **Results:** Among 41 patients (11 male/30 female, mean age 56 [29–70]), 63% had low education and 51% had metabolic syndrome. Good MD adherence stood at 14.6%. Poor adherence tied to diet-induced higher costs (p = 0.021) and difficulty to new-diet adoption (p = 0.026). **Conclusion:** Tunisian NAFLD patients had low MD adherence due to financial constraints and dietary adaptation challenges.

## Plain language summary

The study focused on nonalcoholic fatty liver disease (NAFLD), a widespread liver disease with increasing prevalence. It aimed to assess Tunisian NAFLD patients' adherence to the Mediterranean diet (MD), an essential aspect of NAFLD management that lacked sufficient investigation in this context. Over a 3-month period, a nutritionist introduced and supervised the MD. Adherence was measured using MEDAS scores, with scores of 10 or higher indicating good compliance. Additionally, patients answered six questions about barriers. Only 14.6% of the 41 patients adhered well to the MD. The study underscores that Tunisian NAFLD patients face challenges in adhering to the MD, primarily due to financial constraints and dietary adaptation issues, highlighting the need for tailored support in managing their condition.

## Tweetable abstract

Limited short-term adherence to the Mediterranean Diet in Tunisian NAFLD patients. Major hurdles: extra food costs & adapting to a new diet. Personalized, extended support is the key to better adherence.

#Nutrition #Health #NAFLD #MediterraneanDiet

## Abstract (plain language summary)

Short-term adherence to the Mediterranean diet is low among Tunisian patients with nonalcoholic fatty liver disease.

The primary barriers to achieving strong adherence are the financial burden associated with the Mediterranean diet and the challenges of adapting to a new dietary pattern.

Personalized psychological and nutritional support extended over time could be instrumental in enhancing adherence to the Mediterranean diet.

Nonalcoholic fatty liver disease (NAFLD) represents the hepatic manifestation of a multi-systemic metabolic disorder. It ranks as the foremost cause of chronic liver disease globally, with a growing prevalence. The disease spectrum varies from simple hepatic steatosis to cirrhosis, accompanied by complications like hepatocellular carcinoma [1]. Managing NAFLD primarily involves reducing insulin resistance, curbing oxidative stress and addressing underlying risk factors. Lifestyle modifications, including balanced exercise and a diet of both quality and quantity, constitute vital components of this therapeutic approach [2,3].

Thus, the Mediterranean diet (MD) has been recommended as the preferred dietary choice for managing NAFLD in several clinical practice guidelines [4]. It offers significant benefits for individuals with nonalcoholic fatty liver disease (NAFLD) by improving metabolic health and reducing associated disruptions such as abdominal obesity, high triglyceridemia, hypertension, high fasting glycemia and low HDL-cholesterol. This diet's anti-inflammatory and antioxidant components, found in olive oil, whole grains, fruits, vegetables and fish, help reduce inflammation and oxidative stress. Additionally, polyphenols in staples like olive oil, walnuts and red wine regulate genes and pathways related to these factors. As obesity and Metabolic Syndrome increase NAFLD risk, the Mediterranean diet's benefits extend to NAFLD by reducing weight, insulin resistance and liver fat content. Hence, the Mediterranean diet's effects on metabolism, inflammation and insulin resistance make it a promising strategy for NAFLD management [5]. However, limited knowledge exists regarding its acceptability and adherence among NAFLD patients, especially within the Tunisian population. Therefore, our study aimed to assess adherence to the Mediterranean diet (MD) in Tunisian NAFLD patients and identify barriers to maintaining this diet.

## Materials & methods

### Patients & study design

It was a prospective study carried out in a gastroenterology department of a tertiary hospital from 17 November 2022 to 2 February 2023.

Patients, over the age of 18, diagnosed with NAFLD based on abdominal ultrasound data and presenting to the outpatient department during the study period, were included. Non-consenting patients and those with secondary hepatic steatosis (due to alcohol, hepatitis B, C, hypothyroidism, drugs, Wilson's disease, etc.) were excluded. Patients lost to follow-up at the time of diet adherence assessment or with known psychiatric disorders were excluded.

### Course of the study

#### Our study comprised two consecutive stages

- Stage 1: patients were included after obtaining their consent. We collected socio-demographic and clinical characteristics through interviews and by reviewing the patient's medical file. Following this, a 20–30 min interview with a nutritionist was conducted to explain the principles of the Mediterranean diet;
- Stage 2: a second interview with the nutritionist was scheduled for 1 month after inclusion to assess adherence to the MD and identify any barriers to maintaining this diet. For patients unable to visit the study site, telephone interviews were recommended;
- Adherence to the diet was assessed using the Mediterranean Diet Adherence Screener (MEDAS) questionnaire from the PREDIMED study [6]. This questionnaire consists of 14 questions, with one point allocated for each validated item, resulting in a final adherence score;
  ✓ A score ranging from 0 to 5 points indicated poor adherence to the MD;
  ✓ A score ranging from 6 to 9 points indicated modest adherence to the MD;
  ✓ A score ranging from 10 to 14 points indicated good adherence to the MD;
- At the end of this questionnaire, adherence to the MD was defined as a questionnaire score of ≥10;
- During the interview, we explored barriers to good adherence to the MD through six direct questions posed to the patients. This allowed us to identify the possible presence of the following factors:
  ✓ Poor understanding of the diet
  ✓ Lack of motivation to follow the diet
  ✓ Insufficient time to prepare specific foods for the diet
  ✓ Increased food costs associated with the diet
  ✓ Lack of cooperation from family members
  ✓ Difficulty in adapting to a new diet

### Ethical considerations

The study protocol, processing and data storage were carried out in accordance with the Declaration of Helsinki. Informed written consent was obtained from all patients before their inclusion in the study.

### Statistical analysis

The data were entered and analyzed using SPSS software version 26, developed by IBM (International Business Machines Corporation), a multinational technology company based in USA. For the analytical study, we employed Pearson's Chi-squared test to compare percentages or qualitative variables. When the validity conditions of the Chi-squared test were not met, we utilized Fisher's exact test. In addition to these tests, we conducted univariate analysis to assess specific factors related to adherence. The significance level for all statistical tests was set at 0.05.

## Results

### Study population

41 patients, with an average age of 56 and a sex ratio (M/F) of 0.36, were included.

The main study population characteristics are summarized in Table 1.

**Table 1.** Patient characteristics.

Characteristics	Frequency
**Number**	41
**Age (years)**	56 (29–70)
**Gender (M/F)**	11/30
**Educational level (%)** – Illiterate – Primary – Secondary or university	12 51 37
**Socioeconomic level (%)** – Stockings^†^ – Medium^‡^ – Good^§^	7 88 5
**Professional activity (%)** – Yes – No	39 61
**BMI (kg/m^2^)**	30.74 ± 4.23
**Physical activity (%)** – Sedentary^¶^ – Moderate^#^ – Important^††^	54 14 32
**Comorbidities (%)** – Diabetes mellitus – Arterial hypertension – Dyslipidemia – Overweight (BMI >25 kg/m2) – Metabolic syndrome	41 46 58 95 51

† Average household income is <400 TD.

‡ Average household income between 400 and 1000 TD.

§ Average household income >1000 TD.

¶ Sedentary: Irregular practice or absence of sporting physical activity.

# Moderate physical activity: regular participation in at least 30 min of sporting physical activity one to two-times per week.

†† Important physical activity: regular participation in at least 30 min of sporting physical activity ≥three-times per week.

TD: Tunisian Dinar.

### Adherence to the Mediterranean diet

The mean score of the MEDAS questionnaire was 7.34 ± 1.78 points [^5-12^].

**Figure 1.**
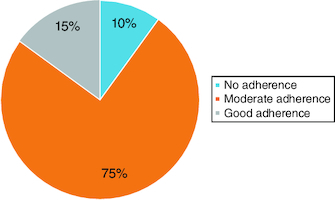
Adherence to the Mediterranean diet after 1 month of initiation.

Good adherence to MD was observed in 15% of patients, while the majority (75%) showed moderate adherence. 10% of patients did not adhere to this diet (Figure 1).

### Barriers to good adherence to the Mediterranean diet

Patients reported that the most significant barriers to adhering to the MD were the additional food costs induced by the diet and difficulties in adapting to a new dietary regimen (Table 2).

**Table 2.** Barriers to good adherence to the Mediterranean diet.

Barrier	Frequency (%)
Misunderstanding of the MD	29
Lack of willpower to follow the MD	24
Lack of time to prepare the specific foods in the MD	7
Extra food cost induced by the MD	50
Uncooperative family members	12
Difficulty of adapting to a new dietary mode	37

MD: Mediterranean diet.

In univariate analysis, non-adherence to the Mediterranean diet was statistically associated with extra food costs induced by the MD (p = 0.021) and with the difficulty of adapting to a new diet (p = 0.026) (Table 3).

**Table 3.** Risk factors for lack of good adherence to the Mediterranean diet.

Factor	Good adherence		p-value
	Yes (n = 6)	No (n = 35)	
Gender (%) M F	33 67	26 74	0.651 0.651
Low or medium socioeconomic level (%)	67	63	1
The illiterate or primary level of education (%)	100	94	1
Misunderstanding of the MD (%)	50	26	0.334
Lack of willpower to follow the MD	0	29	0.307
Lack of time to prepare the specific foods in the MD (%)	0	9	1
Extra food cost induced by the MD (%)	0	57	**0.021**
Uncooperative family members (%)	17	11	0.567
Difficulty of adapting to a new dietary mode (%)	83	31	**0.026**

Bold values indicates risk factors statically associated with non-adherence to the Mediterranean diet.

MD: Mediterranean diet.

## Discussion

Our study highlights the low adherence to the MD among individuals with NAFLD, despite the fact that our study population resides in a mediterranean region. The challenges to achieving good adherence were twofold: first, patients faced difficulty adapting to a new diet; and second, the MD itself posed financial barriers due to additional costs.

To the best of our knowledge, this is the first Tunisian study to focus on this topic. Adherence to the MD was assessed using an objective tool – the MEDAS score – a brief, quantifiable questionnaire comprising 14 questions. This tool is user-friendly, reproducible and less time-consuming compared with traditional dietary assessment methods. It was initially validated in a large multicenter randomized controlled study, the PREDIMED study, conducted in Spain with 7747 patients at high cardiovascular risk adhering to the MD. In this study, the MEDAS score demonstrated a significant and favorable correlation when compared with the classic ‘food frequency questionnaire’ [7]. Subsequently, several authors have employed it to evaluate MD adherence [^8-10^].

MD was first described in Greece and southern Italy in the 1960s by Ancel Keys. It is defined as a diet low in saturated fatty acids and high in vegetable oils [11]. This diet reflects the typical traditional dietary pattern of the Mediterranean region, including Tunisia. However, MD appears to be gradually disappearing from the dietary habits of Mediterranean populations [^12-14^].

MD has demonstrated beneficial effects in several pathologies, including NAFLD, as well as in the prevention of cancers and neurodegenerative disorders, the reduction of cardiovascular risk, all-cause mortality and even in the treatment of depression [6,^15-18^]. In the case of NAFLD, MD improves insulin sensitivity, reduces intrahepatic steatosis and mitigates hepatic fibrosis [5]. However, there is limited knowledge regarding the rate of adherence to MD and the potential obstacles to achieving good adherence to this diet.

The largest series is an Italian study that included a statistically representative sample of the Italian population (36,000 subjects). This study demonstrated that good adherence to the Mediterranean Diet (MD), assessed by the Mediterranean Diet Index, was observed in only 12% of cases [13].

These results are corroborated by studies conducted in Spain [19,20] and Greece [10]. However, it's important to note that the patients included in these studies did not receive dietary education before the assessment of their adherence to the MD. Additionally, the majority of the studied populations, with the exception of the Italian study, consisted of students. It has been shown in previous studies that the diet of younger individuals is transitioning toward a more westernized diet. These trends are also observed in countries outside the Mediterranean region [21].

Furthermore, it was observed that adherence to the diet was notably higher among patients already familiar with NAFLD [22]. However, there has been limited focus on evaluating MD adherence following dietary education. A study conducted in UK, involving 19 patients with NAFLD who received education on MD and had their adherence assessed using the MEDAS score, showed that dietary education significantly improved overall adherence. Participants went from moderate adherence (average score of 7.6 ± 2.5) to high adherence (average score of 9.8 ± 2.8) after 12 weeks of initiating the diet.

It is worth noting that in this study, dietary education included practical support for the diet, such as providing recipes, a meal diary/meal planner and a shopping list. This support likely contributed to the difference between these results and those reported in our study, highlighting the importance of combining such resources with a multifaceted approach that includes modern technologies like mobile applications and cooking workshops to enhance daily adherence to MD [23].

The significant impact of therapeutic education is further emphasized by the results of a Greek randomized controlled trial involving 36 patients with NAFLD. This trial demonstrated that providing dietary education for MD through seven group sessions, each lasting 60 min and spread over 6 months, significantly increased adherence to MD compared with a placebo group that only received an informative brochure (p < 0.05) [3]. The extended duration of education is a notable advantage as it helps solidify MD as a ‘healthy’ lifestyle rather than just an imposed diet.

Despite MD's palatability [24], unlike some other diets like the gluten-free diet, there have been reported barriers to good adherence. These barriers, described in the literature, relate to the socio-demographic characteristics of patients, their lifestyles, motivation and taste preferences [8]. Notably, the additional cost induced by the diet emerged as the most frequently cited obstacle in various studies, which aligns with our findings [14,21,25,26]. The challenge of adapting to a new dietary pattern also remains a significant hurdle [8,21]. These findings underscore the importance of personalized dietary advice that takes into account the patient's socio-economic conditions and offers practical, budget-friendly recipe ideas. Moreover, it highlights the need for extended dietary education, as demonstrated in the study by Katsagoni *et al.* [3], which can provide valuable psychological support and aid patients in better adapting to their new diet.

Male gender, youth, low educational attainment and lower socioeconomic status have been associated with reduced adherence to the Mediterranean diet (MD) [20,27], along with insufficient family support [14], a limited understanding of MD [28], reluctance to embrace a new dietary regimen and resistance to changing typical food preferences [25].

However, it is important to note that these obstacles did not reach statistical significance in our series, likely due to the small sample size. It is worth mentioning that the understanding of MD is closely tied to the patients' education level, as well as the quality of dietary education, as discussed earlier. In our study, a significant proportion of the included patients had limited education, with many being illiterate or having only completed primary education. This educational background could indeed influence adherence to MD and may introduce a selection bias into our findings.

Age was not specifically analyzed in our study, as the average age of our patients was 54 years old. Moreover, Comorbidities had no significant impact on the outcomes since we used the validated MEDAS score, enabling us to assess Mediterranean diet adherence independently of comorbid conditions or medications.

Finally, a lack of understanding regarding the cause and significance of NAFLD has been observed to negatively impact the willingness to make dietary changes [8]. Therefore, it is advisable to actively engage the patient in the management of their disease by providing clear information about NAFLD, its evolving risks, and the expected benefits of adhering to the MD in order to enhance their motivation.

This study sheds light on the challenges of implementing MD, even within a population residing in the Mediterranean region. It underscores the limitations of a single session of traditional dietary education in sustaining strong adherence to MD in the short term. The additional financial burden further compounds the issue, perpetuating suboptimal adherence to MD, especially in low- and middle-income countries, as is evident in our study.

Nevertheless, our study possesses certain limitations, most notably the relatively small sample size. A larger cohort would have enhanced the statistical power and bolstered the reliability of our findings. Another limitation lies in the short duration of the follow-up period (1 month). To offer a more comprehensive view of MD adherence, longer-term evaluations are warranted.

We propose a comprehensive nutritional care approach that involves close collaboration between clinicians and nutritionists to ensure better patient adherence to their prescribed diet. This approach would encompass the following key components:

▪ Thoroughly educating patients about the spectrum of NAFLD, its progression, and potential risks;
▪ Providing a clear explanation of how the Mediterranean Diet (MD) can influence the course of NAFLD;
▪ Tailoring the MD plan to align with the patient's culinary preferences and socio-economic conditions;
▪ Implementing dietary education programs spread over several months, supplemented by resources such as recipe books or mobile applications, to offer ongoing support and help maintain adherence to the diet.

## Conclusion

This study highlights the limited short-term adherence to the MD among Tunisian patients with NAFLD. The primary obstacles to achieving better adherence were the additional food costs and the challenges associated with adapting to a new dietary regimen. To enhance adherence to this diet, personalized and extended psychological and nutritional support should be considered.
